# The effects of school‐based interventions on physiological stress in adolescents: A meta‐analysis

**DOI:** 10.1002/smi.3081

**Published:** 2021-07-26

**Authors:** Amanda W. G. van Loon, Hanneke E. Creemers, Ana Okorn, Simone Vogelaar, Anne C. Miers, Nadira Saab, P. Michiel Westenberg, Jessica J. Asscher

**Affiliations:** ^1^ Department of Child and Adolescent Studies Utrecht University Utrecht The Netherlands; ^2^ Forensic Child and Youth Care Sciences University of Amsterdam Amsterdam The Netherlands; ^3^ Behavioural Science Institute Radboud University Nijmegen The Netherlands; ^4^ Developmental and Educational Psychology Leiden University Leiden The Netherlands; ^5^ Graduate School of Teaching (ICLON) Leiden University Leiden The Netherlands

**Keywords:** adolescents, meta‐analysis, physiological stress, school‐based intervention programs

## Abstract

Chronic stress is associated with dysregulations in the physiological stress system, resulting in diverse negative developmental outcomes. Since adolescence is a period characterized by increased stress‐sensitivity, and schools are an important environment for the developing adolescent, school‐based interventions promoting psychosocial functioning are of particular interest to prevent adverse outcomes. The present study therefore aimed to investigate the effectiveness of such interventions on hypothalamic pituitary adrenal‐axis (i.e., cortisol) and cardiovascular (i.e., blood pressure [BP] and heart rate [HR]/heart rate variability [HRV]) parameters of stress in adolescents, and examined moderators of effectiveness. The search resulted in the inclusion of *k* = 9 studies for cortisol, *k* = 16 studies for BP, and *k* = 20 studies for HR/HRV. The results indicated a significant small overall effect on reducing BP, but no significant effect for HR/HRV. For cortisol, large methodological variation in the few primary studies did not allow for quantitative analyses, but a qualitative review demonstrated inconsistent results. For BP and HR/HRV, larger effects were observed for intervention programs with a mindfulness and/or meditation component, for interventions without a cognitive‐behavioural component and for interventions with a higher intensity. Providing adolescents with techniques to improve indicators of physiological stress may prevent emerging mental health problems.

## 1 INTRODUCTION

Worldwide, stress seems to be a notable problem for children, adolescents, and adults (Klinger et al., 2015; Schaufeli et al., 2009; Valizadeh et al., 2012). Stress is the condition or feeling that results when the demands of a situation exceed the personal, psychological or social resources of an individual (Lazarus, 1966). In adolescence, a period characterized by psychosocial and physiological changes and increased stress‐sensitivity (Romeo, 2013; van den Bos et al., 2014), high levels of stress are associated with various negative outcomes, including reduced academic performance (Arsenio & Loria, 2014; Kaplan et al., 2005; Liu & Lu, 2011) and mental health problems, such as depression and anxiety (de Bruin et al., 2018; Jayanthi et al., 2015; Snyder et al., 2019; Walburg, 2014). In order to prevent adverse outcomes, it is important to address heightened stress levels during adolescence.

Perceived stress often reflects psychological stress, with symptoms such as worrying, exhaustion, and concentration problems (Schraml et al., 2011; Valizadeh et al., 2012). At a physiological level, situations appraised as threatening or challenging, evoke a stress response. Two major systems are responsible for the stress response, respectively the autonomic nervous system (ANS) and the hypothalamic pituitary adrenal (HPA) axis (Charmandari et al., 2005; Romeo, 2013). In response to a stressor, the first system triggers the fight‐or‐flight response through the sympathetic nervous system (SNS) and the adrenal glands, activating involuntary body functions such as breathing, blood pressure (BP), and heartbeat. Physiological changes, including increased systolic blood pressure (SBP), diastolic blood pressure (DBP), heart rate (HR), and respiratory rate (Charmandari et al., 2005; Jezova et al., 2004), ensure that an individual is alert and attentive and can quickly respond to the stressor. After the stressor has passed, the body returns to its normal state through the parasympathetic nervous system (PNS) (Charmandari et al., 2005). Heart rate variability (HRV), which is the variation over time of the period between heartbeats, is an indicator of stress that reflects the balance between sympathetic and parasympathetic activity (Acharya et al., 2006). The second (slower) system (i.e., the HPA axis), regulates the release of the hormone cortisol that influences many bodily functions, including the immune system, the body's metabolism, and the regulation of mood and emotions (Tsigos & Chrousos, 2002), necessary for responding to stressful situations (Nesse et al., 2016). Chronic stress can dysregulate the stress system by persistently activating the SNS and HPA‐axis, causing the body to stay in a constant state of alertness (i.e., ‘fight‐or‐flight response’) (Charmandari et al., 2005). Moreover, as a result of chronic stress, the negative feedback mechanism that controls the stress response fails to work. Consequently, the stress‐induced production of cortisol is not stopped, causing levels of physiological stress to remain high (Mariotti, 2015). As a dysregulated stress system results in diverse negative consequences, including metabolic, autoimmune, cardiovascular, and psychiatric disorders (Charmandari et al., 2005; Miller & O'Callaghan, 2002), it is important to intervene at an early stage to improve ANS and HPA‐axis functioning.

Various intervention programs have been developed to address heightened stress levels in adolescents, offering different approaches to reduce stress. Some of these programs target stress reduction directly, whereas others address stress reduction as an indirect or secondary program target. Often used approaches include mindfulness, meditation, relaxation exercises, and cognitive‐behavioural techniques (Rew et al., 2014). Although these approaches are commonly used as a form of stress management, the neurobiological effects of such programs are still not clearly understood (Heckenberg et al., 2018; Pascoe & Bauer, 2015; Pascoe & Crewther, 2016; Pascoe, Thompson, Jenkins, et al., 2017; Pascoe, Thompson, & Ski, 2017). Recent reviews in diverse populations – community and clinical samples involving youth and adults – showed that mindfulness, meditation, and yoga interventions can improve HPA‐axis and cardiovascular parameters of stress, manifested in reductions in cortisol, HR, SBP, and DBP (Heckenberg et al., 2018; Pascoe & Bauer, 2015; Pascoe & Crewther, 2016; Pascoe, Thompson, Jenkins, et al., 2017; Pascoe, Thompson, & Ski, 2017) and increased HRV (Heckenberg et al., 2018; Pascoe, Thompson, & Ski, 2017). Yet, only a limited number of studies targeted adolescents, who are particularly vulnerable to stress and the adverse effects of prolonged stress (Romeo, 2013), and may, therefore, be an important target group. In particular, school‐based intervention programs are of interest to examine, as schools are an important environment for adolescents' cognitive, social, and emotional development (Roeser et al., 2000), and provide a promising domain for low‐threshold care (Stephan et al., 2007). Previous reviews demonstrated that school‐based intervention programs reduced stress (Kraag et al., 2006; Rew et al., 2014; van Loon et al., 2020). However, these reviews primarily focused on psychological stress. Overall, a synthesis on the effects of intervention programs on physiological parameters of stress is lacking, in particular for school‐based intervention programs in adolescents. Moreover, when examining the effectiveness of school‐based intervention programs on stress, physiological stress should not be ignored given the negative consequences of a dysregulated stress system (Charmandari et al., 2005; Miller & O'Callaghan, 2002), including mental health problems such as anxiety and depression (Romeo, 2013). The current study, therefore, aims to investigate the effectiveness of school‐based intervention programs promoting psychosocial functioning on indicators of physiological stress in adolescents.

Previous research demonstrated that various characteristics influenced the effectiveness of school‐based intervention programs on stress outcomes, including type of intervention (Feiss et al., 2019; van Loon et al., 2020) and study quality (Kraag et al., 2006). In order to detect which interventions or components are most effective in improving HPA‐axis and cardiovascular parameters of stress and which subgroups benefit the most (Kraemer et al., 2002), it is important to investigate moderators. We therefore selected several intervention, sample, and study characteristics as moderators of program effectiveness, based on previous meta‐analyses investigating the effects of intervention programs in adolescents.

In terms of intervention characteristics, program intensity might moderate the effectiveness, since larger effects on depressive symptoms have been found for depression prevention programs with shorter durations (i.e., less than 12 h) (Stice et al., 2009). Furthermore, content of the intervention might moderate effectiveness, as larger effects on stress outcomes have been found for school‐based programs that taught problem solving and emotional coping skills compared to relaxation techniques (Kraag et al., 2006).

In addition, several sample characteristics could influence the effectiveness of school‐based programs on physiological stress, including age, gender, ethnicity, and type of intervention. Larger effects for depressive symptoms have been found for samples with older adolescents, a higher proportion of females, and more participants from ethnic minorities (Stice et al., 2009), and for targeted and selected high‐risk samples as opposed to non‐selected, community samples (Feiss et al., 2019; Stice et al., 2009; van Loon et al., 2020).

Lastly, study characteristics might moderate the effectiveness, for instance publication year, since recent publications are more likely to report null‐results (Kaplan & Irvin, 2015). Moreover, in earlier meta‐analyses examining the effects of intervention programs in adolescents, larger effects were observed for lower versus higher quality studies (Kraag et al., 2006), for studies with quasi‐experimental designs compared to randomized controlled trials (RCTs) (Suter & Bruns, 2009), for active versus passive control groups (Feiss et al., 2019), and for follow‐up compared to post‐intervention measurements (van Loon et al., 2020), demonstrating that these characteristics require attention.

In the present study, we aimed to investigate the effectiveness of school‐based intervention programs on HPA‐axis and cardiovascular parameters of stress in adolescents. In particular, we examined the effects on cortisol, BP (i.e., SBP and DBP), and HR/HRV (i.e., HR, pulse rate and HRV [low frequency (LF), high frequency (HF) and coherence]). We expected that school‐based intervention programs would reduce cortisol, BP, and HR (Heckenberg et al., 2018; Pascoe & Bauer, 2015; Pascoe, Thompson, Jenkins, et al., 2017; Pascoe, Thompson, & Ski, 2017), while HRV was expected to increase (Heckenberg et al., 2018; Pascoe, Thompson, & Ski, 2017). In addition, we examined which intervention (i.e., components, intensity), sample (i.e., target group, gender, minority background, and age), and study characteristics (i.e., type of HR and BP measurement, type of stress outcome, publication year, study design, type of comparison condition, timing of outcome measurement, and study quality) moderate the effectiveness.

## 2 METHODS

### 2.1 Selection criteria

In the current study, studies were included if the following inclusion criteria were met: (1) studies evaluating the effectiveness of a school‐based intervention program promoting psychosocial functioning (including stress reduction, mental health or coping skills), (2) studies had at least one physiological stress‐related outcome (i.e., cortisol, SBP, DBP, HR, pulse rate or HRV), (3) studies had to target adolescents (sample between 10 and 18 years old at the start of the intervention), (4) studies had to compare an experimental group with a control group, (5) studies had to have a pre‐intervention physiological measurement and a post‐ and/or follow‐up physiological measurement in one of the domains of interest (i.e., cortisol, BP, HR or HRV), (6) the manuscript had to be written in English, and (7) studies had to report sufficient statistics for performing a meta‐analysis. Studies evaluating an intervention that primarily focused on improving physical health and only aimed to promote psychosocial functioning by improving physical health through physical activity or exercise were excluded.

### 2.2 Search strategy

Relevant publications were identified using the search engines Cumulative Index to Nursing and Allied Health Literature (CINAHL), PubMed, Education Resources Information Center (ERIC), PsycINFO, and Cochrane Central Register of Controlled Trials (CENTRAL), with the search period up until May 2020. The search strings were ‘intervention* or program*’ in combination with ‘stress or autonomic nervous system or ANS or sympathetic nervous system or SNS or parasympathetic nervous system or PNS or hypothalamus‐pituitary‐adrenal axis or hypothalamic pituitary adrenal axis or HPA‐axis or HPA axis or cortisol or blood pressure or pulse rate or heart rate or variability’ in combination with ‘adolesc* or child or children or youth’ in combination with ‘controlled clinical trial or controlled trial or clinical trial or random* or experiment* or comparison group* or controls or control condition* or control group* or control subject* or no treatment group* or waiting list or wait list or waitlist or treatment as usual or care as usual’ in combination with ‘school*’. Additionally, not statements (i.e., oxidative stress, distress syndrome, parenting stress, immunization, vaccination, venipuncture, animals, infants, toddlers, preschool, kindergarten, pregnancy, neonatal, prenatal, study protocols, reviews and meta‐analyses) were used to exclude studies that did not fit the inclusion criteria. Search hits were subjected to a first screening based on title and abstract, performed by the first author. Hits that were deemed eligible for inclusion, were subjected to a second screening, based on full‐text, by the first author. A subset of hits of the first and second screening (13%) was also screened by the third author, which revealed no discrepancies. Google Scholar was used to check the first 100 hits for grey literature (i.e., unpublished research) and missing relevant publications, and we conducted a manual search through the reference lists of the included publications and relevant reviews (Feiss et al., 2019; Kraag et al., 2006; Pascoe & Bauer, 2015; Pascoe & Crewther, 2016; Pascoe, Thompson, Jenkins, et al., 2017; Pascoe, Thompson, & Ski, 2017; Rew et al., 2014; van Loon et al., 2020). If a manuscript that seemed relevant did not provide the statistics needed to calculate an effect size or did not report data of interest, authors were contacted to provide the missing information.

### 2.3 Coding of studies

Study characteristics, outcome variables, and moderators were registered using a detailed coding sheet. All studies were coded by the first author. The third author coded a subsample of studies (i.e., double coded studies) and responses of both researchers were compared (inter‐rater reliability was 87% for a subsample of 13% of the studies). Effect sizes were coded for three categories of parameters of physiological stress: cortisol (reflecting HPA‐axis functioning), BP (i.e., SBP and DBP, reflecting ANS functioning) and HR/HRV (i.e., HR, pulse rate, and HRV, reflecting ANS activity). We included studies that investigated basal functioning (i.e., measurements during a resting period), ambulatory monitoring (i.e., 24‐h measurements at regular intervals reflecting activity of everyday life), and stress reactivity (i.e., responses to a stress inducing task, including stress reactivity and recovery). Stress reactivity was based on difference scores (Allen et al., 2014; Linden et al., 1997), calculated by subtracting pre‐stressor or baseline levels from levels during anticipation and/or performance of the stress task. Stress recovery was calculated by subtracting levels during performance of the stress task from recovery levels (i.e., after completion of the stress task) (Kudielka et al., 2004). A reduction from baseline to post‐intervention or follow‐up measurement reflected an improvement for the parameters cortisol, SBP, DBP, HR, and pulse rate, while an increase from baseline to post‐intervention or follow‐up measurement reflected an improvement for the parameter HRV (i.e., HRV and the components LF, HF, and coherence), for both basal functioning and ambulatory monitoring, as well as stress reactivity. HF HRV reflects PNS activity, while overall HRV, coherence, and LF reflect both sympathetic and parasympathetic activity. An increased HRV therefore indicates a more balanced and coherent (i.e., healthy) system (Acharya et al., 2006; McCraty et al., 2009).

The following characteristics were coded as moderators (i.e., intervention, sample and study characteristics).

*Intervention characteristics* were whether or not the intervention included specific stress reduction components, that is, mindfulness and/or meditation (yes or no), relaxation exercises (yes or no), yoga (yes or no), and cognitive‐behavioural techniques (yes or no), and intensity of the intervention (session duration multiplied by the frequency of sessions, analysed as a continuous variable).

*Sample characteristics* were target group (non‐selected vs. selected student samples, with selected students defined as included based on self‐selection or screening), percentage of boys (as a continuous variable), percentage of minorities (i.e., non‐Caucasian, analysed as a continuous variable), and mean age of the adolescents (as a continuous variable).

*Study characteristics* were type of HR measurement (HR or pulse rate vs. HRV), type of BP measure (SBP vs. DBP), type of stress outcome (basal functioning, stress reactivity, or ambulatory monitoring), publication year, study design ((cluster) RCT or quasi‐experimental study), type of comparison condition (passive control vs. active control), timing of outcome measurement (at post‐intervention or at follow‐up), and study quality (as a continuous variable). Study quality was assessed using the Quality Assessment Tool for Quantitative Studies (Thomas et al., 2004). Six variables were used, including selection bias, study design, confounders, blinding, validity of data collection methods, and withdrawals and dropouts. Using these variables – each scored with 0 (missing/not accounted for), 1 (somewhat accounted for) or 2 (accounted for) – a total study quality score was calculated (with a range of 0‐12 per study).

### 2.4 Analysis of effect sizes

An online effect size calculator (Wilson, n.d.) was used to calculate Cohen's *d*'s for each effect size, indicating the effectiveness of school‐based intervention programs on physiological parameters of stress, based on the difference between adolescents receiving an intervention program relative to adolescents in a control group. An improvement favouring the intervention group over the control group was expressed in a positive effect size. Both pre‐ and post‐intervention or follow‐up measurement group differences were computed and pre‐measurements *d*'s were subtracted from post‐intervention or follow‐up measurement *d*'s to take into account baseline differences between groups (e.g., van der Stouwe et al., 2014). For instance, a larger reduction in BP from baseline to post‐intervention for the intervention group compared to the control group reflects intervention effectiveness and is expressed in a positive Cohen's *d*. Conversely, for HRV, a larger increase from baseline to post‐intervention for the intervention group compared to the control group reflects intervention effectiveness (i.e., expressed in a positive Cohen's *d*). For most of the cases, Cohen's *d* was calculated based on means and standard deviations (*SD*s) or mean difference scores. When those were not reported (35.8% of the total number of effect sizes), Cohen's *d* was calculated based on least‐square or marginal means and standard errors (*SE*). Some effect sizes were calculated on transformed data, including log transformations, slopes, and area under the curve (9.9% of the total number of effect sizes). According to Cohen (1988), a small effect size was considered *d* = 0.20, a moderate effect size *d* = 0.50, and a large effect size *d* = 0.80. For categorical moderators dummy variables were computed, and continuous moderators were centred around their mean.

Overall effect size and moderator analyses were conducted with a three‐level meta‐analytic model in *R* (Assink & Wibbelink, 2016), taking into account the dependency of multiple effect sizes from one study (van den Noortgate et al., 2013). The first level included the sampling variance of each effect size (level 1), the second included the within‐study variance of effect sizes of the same study (level 2), and the third included the between‐study variance of effect sizes from different studies (level 3). An intercept‐only model was used to estimate the overall effect. Extreme effect sizes (interquartile range > 3) (Elbaum et al., 2000) were adjusted by winsorizing them (i.e., replacing the outlier by the lowest or highest score within the normal range) (Spruit et al., 2020). Significant heterogeneity was assessed by performing log‐likelihood tests on level 2 (variance within studies) and level 3 (variance between studies). Moderator analyses were performed if there was significant heterogeneity for at least one of the levels, by including the potential moderators in the three‐level model (Assink & Wibbelink, 2016).

### 2.5 Publication bias

When conducting meta‐analyses, it is important to take into account the influence of publication bias (i.e., studies with positive results are more likely to be published compared to studies with negative or non‐significant results). We examined indicators of publication bias by calculating the fail‐safe *N* (i.e., if the fail‐save *N* exceeds the critical value, calculated by the formula 5 *×* *k* + 10, no publication bias is indicated) (Rosenthal, 1979), by visually exploring funnel plots, and conducting a multilevel analysis with the sampling variance as a moderator. In addition, we performed trim and fill analyses to examine asymmetry and the effect of missing effect sizes on the results (Duval & Tweedie, 2000a, 2000b).

## 3 RESULTS

### 3.1 Study selection

**FIGURE 1:**
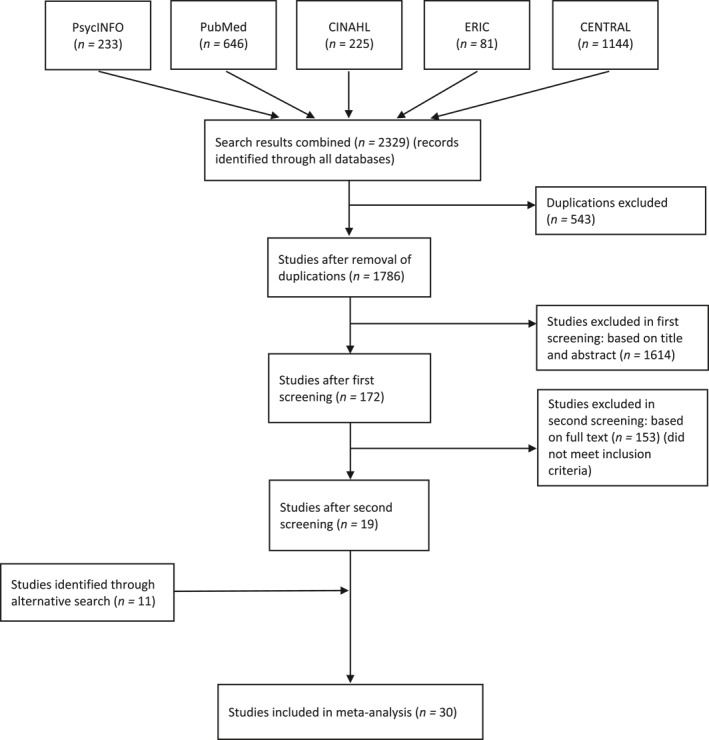
Flow chart

As displayed in the flowchart (Figure 1), the electronic search identified 2329 unique hits for all databases after the removal of duplicates. After first selection by screening the title and abstract of the publications, 172 studies were potentially eligible. After full text screening, 19 studies met the inclusion criteria. Seven additional manuscripts seemed relevant, but were excluded because they did not include sufficient statistics to calculate an effect size and authors could not provide the missing data. Of the 19 included studies, authors from five studies provided additional information. The alternative search yielded 11 additional studies, which resulted in a final number of *k* = 30 included studies and *N* = 162 effect sizes for all physiological stress outcomes, based on a total of *N* = 4460 participants, with *N* = 2634 participants in the intervention group and *N* = 1826 participants in the control group. Details of the selected studies are provided in Table 1.

**TABLE 1:** Detailed description of the selected studies

Reference	*N*	Mean age, % boys, ethnicity^a^	Intervention	Comparison group	Study design	Target group	Stress outcomes	Significant differences^b^
Barnes et al. (2001)	33	16.6, 54% boys, 97% minorities	Transcendental meditation (*n = *15)	Lifestyle education (*n = *18)	RCT	Screened (high SBP)	Resting SBP, DBP, HR (supine position, relaxed for 15 min [three measurements]) SBP, DBP, HR (during 10‐min car‐driving and social stressor [readings every other minute])	Lower resting SBP, decreases in SBP and HR reactivity (car stressor) and SBP reactivity (social stressor) at post‐intervention
Barnes, Treiber, and Johnson (2004)	100	16.2, 63% boys, 100% minorities	Transcendental meditation (*n = *50)	Lifestyle education (*n = *50)	Cluster RCT	Screened (high SBP)	Ambulatory SBP, DBP, HR (daytime, 6 AM to 11 PM [every 20 min] and night‐time, 11 PM to 6 AM [every 30 min])	Decrease in daytime SBP at post‐intervention and follow‐up
Barnes, Davis, et al. (2004)	73	12.3, 53% boys, 52% minorities	Concentration‐type meditation (*n = *34)	Health education (*n = *39)	Cluster RCT	Community	Resting SBP, DBP, HR (recordings for 10 min [average of last three readings]) ambulatory SBP, DBP, HR (daytime school, 8 AM to 3 PM [every 20 min], daytime after school, 3 PM to 10 PM [every 20 min] and night‐time, 12 PM – 6 AM [every 30 min])	Decreases in resting SBP, ambulatory daytime after school SBP, DBP and HR at post‐intervention
Barnes et al. (2012)	170	15.6, 46% boys, 91% minorities	Williams LifeSkills (*n = *91)	Health education (*n = *79)	Cluster RCT	Community	Ambulatory SBP, DBP (daytime school, 8 AM to 3 PM [every 30 min], daytime after school, 3 PM to 10 PM [every 20 min], and night‐time, 10 PM to 6 AM [every 20 min])	None
Bayne‐Smith et al. (2004)	439	16.1, 0% boys, 89% minorities	Physical activity and teenage health (*n = *310)	Traditional PE (*n = *129)	RCT	Community	Resting SBP, DBP (two measurements after 5–15 min of rest in seated position [only second reading recorded])	Reduced SBP and DBP at post‐intervention
Benson et al. (1994)	37	15.5	Health curriculum (*n = *21)	Waitlist (*n = *16)	RCT	Community	SBP, DBP, HR (resting baseline and during MAT)	None
Bradley et al. (2010)	98	15.3, 47% boys, 52% minorities	Heart rhythm coherence biofeedback training (*n = *50)	Waitlist control (*n = *48)	Cluster RCT	Community	Resting HR, HRV (HF power, LF power, coherence) [continuous recordings over a 4‐min resting period] HR, HRV (HF power, LF power, coherence) [continuous recordings over a 4‐min stress preparation period, before SCWC task]	Reduced HR and increased HRV measures at post‐intervention
Calvete et al. (2019)	503	Grade 8 (13.6), 9 (14.6), 10 (15.8), 52% boys	Incremental personality theory (grade 8 *n = *87; grade 9 *n = *132; grade 10 *n = *44)	Educational control (grade 8 *n = *77; grade 9 *n = *125; grade 10 *n = *38)	RCT	Community	Salivary cortisol (sample collected at mean time of 11:02 AM)	None (only pre‐ and two follow‐up measurements)
Chang et al. (2013)	67	12.5, 51% boys	Laughing Qigong (*n = *34)	Read books or did homework (*n = *33)	Quasi‐experiment	Community	Salivary cortisol (cotton wads in mouth for 2 min) Resting HR, HRV (HF, LF), SBP, DBP (measured in supine position)	None
Ewart et al. (1987)	110	14.7, 60% boys, 61% minorities	Progressive muscle relaxation (*n = *51)	Assessment only (*n = *59)	RCT	Screened (high BP)	Resting SBP, DBP (measurements after at least 10 min rest [average of nine measurements over 20‐min period])	Reduced SBP at post‐intervention
Fishbein et al. (2016)	69	16.7, 46% boys, 91% minorities	Mindful yoga (*n = *30)	Regular classes (*n = *39)	RCT	Self‐selected	HR, HRV (pre‐stressor rest, baseline during SCT with no tone, during SCT with tone, recovery period)	None
Flores (1995)	49	12.6, 0% boys, 87% minorities	Dance for health + health education (*n = *26)	Usual PA (*n = *23)	Cluster RCT	Community	Resting HR	Reduced resting HR at post‐intervention
Gregoski et al. (2011)^c^	166	15.0, 41% boys, 100% minorities	Breathing awareness meditation (*n = *53); life skills training (LST) (*n = *69)	Health education control (*n = *44)	Cluster RCT	Screened (high SBP)	Ambulatory SBP, DBP HR (daytime school, 7 AM to 3 PM [every 30 min], after school, 3 PM to 10 PM [every 20 min], and night‐time, 12 AM to 7 AM [every 30 min])	Decreased daytime school and night‐time SBP (BAM) and increased daytime school HR (LST) at post‐intervention
Hagins et al. (2013)	30	10.8, 57% boys, 50% minorities	Yoga (*n = *15)	PE (*n = *15)	RCT	Community	SBP, DBP, HR (two successive measurements after 5 min rest [mean initial rest], at halfway point and at the end of MAT or MTT [end of stressor 1 and 2], and recovery values at the end of 5 and 10 min of rest [mean recovery rest])	None
Killen et al. (1988)	1130	15.0, 100/0% boys, 31% minorities	Special intervention (boys *n = *345, girls *n = *267)	Control (boys *n = *277, girls *n = *241)	Cluster RCT	Community	Resting SBP, DBP, HR (three measurements at 1‐min intervals after sitting quietly for 3 min [means of the second and the third measurement])	Reduced resting HR and increased DBP for boys and girls at follow‐up (only pre‐ and follow‐up measurements)
Lindblad et al. (2007)	43	12.0, 47% boys	Music education (*n = *16)	Normal curriculum (*n = *27)	Quasi‐experiment	Community	Saliva cortisol (collected by swabs, taken at awakening, 30 min after awakening, 1 h after lunch, and before going to bed in the evening) [means log of saliva cortisol]	None
Markham (2004)	66	14.6, 73% boys, 25% minorities	Positive emotional refocusing (*n = *41)	Waitlist (*n = *25)	RCT	Community	Resting HRV (coherence, HF, LF) (baseline period of 7 min [pre‐stressor])	None
McClendon and Scott (2018)	22	14.8, 23% boys, 100% minorities	Yoga (*n = *11)	Indoor track (*n = *11)	Quasi‐experiment	Community	Resting HR, SBP, DBP (prior to the start of any PA)	None
Ørntoft et al. (2016)	494	11.1, 49% boys	FIFA 11 for health (*n = *354)	PE (*n = *140)	Cluster RCT	Community	Resting SBP, DBP (measurements following at least 10 min of rest [average of three measurements]); resting HR (measurements following at least 10 min of rest, 15 s intervals over entire resting period [lowest HR value])	Decreased SBP at post‐intervention
Osborne et al. (2007)	22	13.9, 39% boys	Music performance enhancement program (*n = *13)	Behaviour exposure only (*n = *9)	RCT	Screened (music anxiety)	HR (5 min prior to the start [pre‐stressor], during performance (start and end), and ending 5 min after musical performance [recovery period], recorded continuously)	None
Salazar (2017)	95	15.8, 32% boys, 23% minorities	Mindfulness (*n = *40)	Regular PE (*n = *55)	Quasi‐experiment	Self‐selected	Resting PR (wait 30 s to receive a PR reading)	Lower PR at post‐intervention and follow‐up
Schonert‐Reichl et al. (2015)	99	10.2, 56% boys	Mindfulness‐based education school‐emotional learning (*n = *48)	Social responsibility program (*n = *51)	Cluster RCT	Community	Salivary cortisol (dental cotton roll in mouth for 1 min, collected three times within 1 day at 9 AM, 11:30 AM and 2:30 PM, relative to awakening [slope difference scores − cortisol change across the day])	Improved stress physiology at post‐intervention
Sibinga et al. (2013)	30	12.5, 100% boys, 95% minorities	Mindfulness‐based stress reduction (*n = *19)	Health education (*n = *11)	RCT	Community	Salivary cortisol (collected at two successive days at awakening, 60 min post‐awakening, 2:30 PM, and bedtime [AUC_g_ approach])	None
Sieverdes et al. (2014)	28	12.3, 43% boys, 43% minorities	Hatha yoga (*n = *14)	Music and art classes (*n = *14)	RCT	Community	Resting HR (rest for 10 min [four readings]);resting SBP, DBP (rest for 10 min [averaging last three readings]) salivary cortisol (bedtime, upon awakening, before leaving the bed and 30 and 60 min afterwards [AUC analysis])	None
Skoradal et al. (2018)	392	11.1, 53% boys	FIFA 11 for health (*n = *292)	Normal curriculum (*n = *100)	Cluster RCT	Community	Resting SBP, DBP (assessments after 15‐20 min rest [average of three consecutive measurements]) resting HR (assessments after 15–20 min rest [lowest HR recording])	Decreased SBP at post‐intervention
Telles and Srinivas (1998)	24	14.1	Yoga (*n = *12)	PA (gardening) (*n = *12)	Cluster RCT	Screened (impaired vision)	Resting HR (assessments for 10 min after an initial 15‐min period of rest [counting the QRS complexes in successive 60‐s epochs, continuously])	None
Tomette (2015)	17	16.0, 36% boys, 63% minorities	Healthy living (*n = *11)	Chemistry course (*n = *6)	Quasi‐experiment	Self‐selected	Salivary cortisol	Increased salivary cortisol at post‐intervention
Weigensberg et al. (2009)	12	16.0, 50% boys, 100% minorities	Interactive guided imagery (*n = *6)	Nonintervention control group (*n = *6)	RCT	Screened (overweight)	Salivary cortisol (cotton swab in mouth for 2 min [samples collected at beginning (4 PM) and end of each session (5.30 PM)])	Decreases in salivary cortisol from pre‐ to post sessions
Wright et al. (2011)^c^	121	15.0, 41% boys, 100% minorities	Breathing awareness meditation (*n = *35), life skills training (*n = *42)	Health education (*n* = 44)	Cluster RCT	Screened (high SBP)	Ambulatory SBP, DBP, HR (24‐h)	None (only pre‐ and follow‐up measurements)
Yoo et al. (2016)	42	10.0, 48% boys	Mind‐subtraction meditation (*n = *23)	Reading sessions (*n = *19)	Quasi‐experiment	Community	Salivary cortisol (collection during afternoon hours of 2 to 4 [within 1‐min intervals, participants spat three times in cups])	Lowered salivary cortisol at post‐intervention

Abbreviations: AUC, area under the curve; AUCg, area‐under the curve with respect to ground; DBP, diastolic blood pressure; HF, high frequency; HR, heart rate; HRV, heart rate variability; LF, low frequency; MAT, mental arithmetic task; MTT, mirror tracing task; PA, physical activity; PE, physical education; PR, pulse rate; RCT, randomized controlled trial; SBP, systolic blood pressure; SCT, stroop‐change task; SCW, stroop colour‐word task; VLF, very low frequency.

^a^ Percentage of minorities (i.e., non‐Caucasian).

^b^ Significant differences reported in the included studies (favouring the intervention group over the control group).

^c^ Articles have overlapping samples (i.e., in analyses defined as same sample), sample size of Wright et al. (2011) is not included in the total sample size.

### 3.2 Characteristics of the included studies investigating cortisol

Cortisol was reported in *k* = 9 studies with *N* = 17 effect sizes. Included studies were issued between 2007 and 2019 and only one study was not published (i.e., dissertation). The average age ranged between 10.0 and 16.0 years with a mean age of 13.2 years. The mean percentage of boys was 53.4% and the mean percentage of minorities was 75.3% (based on 4 studies). The study quality ranged from 1 to 10, with a mean score of 6.5. The intervention programs had an average duration of 10 weeks, and the intensity of the programs ranged from 60 to 4040 min (with a mean of 1040 min). All studies measured cortisol during a resting period (no studies with a stress inducing task or ambulatory monitoring). Most studies had a cognitive‐behavioural component (*n* = 6), a relaxation component (*n* = 5) or a mindfulness/mediation component (*n* = 4) in their intervention, while only two studies included a yoga component. Few studies reported that the intervention included homework (*n* = 2), the other studies researched interventions that did not include homework or did not report about this intervention characteristic (*n* = 7). Two studies were performed in selected student samples (one based on screening and one based on self‐selection), the other studies were performed in non‐selected community samples (*n* = 7). Cortisol was measured and analysed in various ways. Some studies collected cortisol samples only once a day (*n* = 5), while others collected samples multiple times a day (*n* = 4), for instance at awakening, after awakening, and bedtime. The timing of the measurements also differed, *n* = 4 studies collected samples that were taken in the morning (after waking up), *n* = 2 studies during lunchtime, *n* = 5 studies in the afternoon, and *n* = 3 studies in the evening (before going to bed). Two studies did not give information about the timing of sample collection. Researchers used cotton wads/swabs to measure cortisol (*n* = 4), let students spit in cups (*n* = 2) or vials (*n* = 2), or did not provide information (*n* = 1). Furthermore, different ways to analyse the data were reported, including using means and *SD*s or *SE*s of cortisol levels (*n* = 5), area under the curve analyses (*n* = 2), log transformations (*n* = 1), or slope difference scores (*n* = 1).

Because cortisol measurements varied considerably across the few included cortisol studies (*k* = 9), in terms of sample collection (e.g., cotton swabs, spitting in cups), number of samples, timing of sample collection (e.g., morning, afternoon), and analytic approach (e.g., area under the curve, slopes), it was not considered meaningful to quantitatively analyse the cortisol findings. Hence, we did not conduct a multilevel meta‐analysis for cortisol, but instead provided an overview of study findings.

### 3.3 Characteristics of the included studies investigating BP

Blood pressure was reported in *k* = 16 studies and *N* = 84 effect sizes. Included studies were issued between 1987 and 2018 and all studies were published (i.e., no dissertations). The average age ranged between 10.8 and 16.6 years with a mean age of 14.3 years. The mean percentage of boys was 48.8% (based on 80 effect sizes) and the mean percentage of minorities was 78.7% (based on 74 effect sizes). The study quality ranged from 3 to 11, with a mean score of 8.4. The intervention programs had an average duration of 11 weeks, and the intensity of the programs ranged from 360 to 3360 min (with a mean of 1500 min). Some studies reported that the researched intervention included homework (*n* = 8), the other studies focused on interventions that did not include homework or did not report about this intervention characteristic (*n* = 12). Only five studies selected students based on screening, and included students with a high BP (four studies specifically focused on high SBP). The other studies (*n* = 11) were performed in non‐selected community samples.

### 3.4 Characteristics of the included studies investigating HR/HRV

Heart rate/HRV was reported in *k* = 20 studies and *N* = 61 effect sizes. Included studies were issued between 1988 and 2018 and only two studies were not published (i.e., dissertations). The average age ranged between 10.8 and 16.7 years with a mean age of 14.4 years. The mean percentage of boys was 48.2% (based on 58 effect sizes) and the mean percentage of minorities was 69.3% (based on 48 effect sizes). The study quality ranged from 1 to 11, with a mean score of 6.7. The intervention programs had an average duration of 12 weeks, and the intensity of the programs ranged from 210 to 3360 min (with a mean of 1400 min). Most studies reported that the intervention included homework (*n* = 10), the other studies focused on interventions that did not include homework or did not report about this intervention characteristic (*n* = 6). The majority of the studies (*n* = 12) were performed in non‐selected community samples, samples of the other studies included students based on self‐selection (*n* = 2) or screening based on high SBP (*n* = 4), impaired vision (*n* = 1) or music anxiety (*n* = 1).

### 3.5 Overall effects

Based on review, overall effectiveness of school‐based intervention programs on cortisol was not indicated. Results of the primary studies for cortisol were inconsistent. On the one hand, three studies demonstrated that school‐based intervention programs were effective in improving cortisol at post‐intervention, indicated by lowered cortisol levels (i.e., measured once in the afternoon in both studies) and a steep slope diurnal pattern (i.e., measured multiple times during the day). On the other hand, one study demonstrated increased mean cortisol levels at post‐intervention (i.e., measured once). Five studies showed that school‐based intervention programs were not effective in improving cortisol levels at post‐intervention and follow‐up (i.e., two studies measured cortisol once, two studies measured cortisol multiple times a day and one study measured cortisol multiple times over two days).

The multilevel meta‐analyses for the effectiveness of school‐based intervention programs on BP yielded a significant yet small overall effect (*d* = 0.173, *SE* = 0.069, *p* = 0.014). Overall, school‐based intervention programs were effective in reducing BP. Five outliers were winsorized. There was significant within‐study variance (*p* < 0.0001), demonstrating heterogeneity.

The overall effect size for HR/HRV was not significant (*d* = 0.134, *SE* = 0.105, *p* = 0.209). Three outliers were winsorized. There was significant within‐study (*p* < 0.0001) and between‐study variance (*p* < 0.05), demonstrating heterogeneity. The overall effect sizes are reported in Table 2.

**TABLE 2:** Result for the overall mean effect sizes

Outcome	*N s*tudies	*N* ES	*N* participants	Mean *d* (*SE*)	95% CI	*t*‐value	LRT	% var	Fail‐safe *N* (cv)
Blood pressure	16 (16)	84	3291	0.173 (0.069)	0.035 to 0.311	2.498*	Level 2: 215.58***	Level 1: 11.3%	2117 (90)
							Level 3: 0.20	Level 2: 85.5%	
								Level 3: 3.2%	
Heart rate	20 (20)	61	2995	0.134 (0.105)	−0.077 to 0.344	1.270	Level 2: 159.98***	Level 1: 11.3%	281 (110)
							Level 3: 5.32*	Level 2: 69.7%	
								Level 3: 18.9%	

Abbreviations: CI, confidence interval; Fail‐safe *N* (cv), fail‐safe number and Rosenthal's critical value in parentheses; LRT, likelihood‐ratio test for level 2 and level 3; mean *d*, mean effect size Cohen's *d*; *N* ES, number of effect sizes; *N* studies (samples), number of studies and independent samples; *SE*, standard error; *t*‐value, difference in mean *d* with zero; % var, percentage of variance explained.

*p < 0.05; **p < 0.01; ***p < 0.001.

### 3.6 Sensitivity analyses

To account for the possible influence of specific studies or effect sizes, we repeated the meta‐analyses after removal of potential influences. First, we repeated the meta‐analyses after removal of the stress recovery effect sizes for BP and HR/HRV, as stress recovery is less clearly understood in relation to stress reactivity (i.e., there is no consensus on how to operationalize stress recovery as indicator of stress) (Linden et al., 1997). Results yielded similar results for BP (*d* = 0.234, *SE* = 0.064, *p* < 0.001) and HR/HRV (*d* = 0.153, *SE* = 0.112, *p* = 0.179). Second, we repeated the meta‐analyses after removal of eight reactivity effect sizes (for both BP and HR/HRV). These effect sizes were generated using baseline or recovery levels that were also used to calculate another effect size in the same study, and were excluded to account for overlap between effect sizes within studies. Results were similar for BP (*d* = 0.209, *SE* = 0.063, *p* = 0.001) and HR/HRV (*d* = 0.141, *SE* = 0.117, *p* = 0.232).

### 3.7 Publication bias

For BP, the Rosenthal fail‐safe test demonstrated there was no indication of publication bias. However, the funnel plot appeared to be slightly asymmetrical and the regression analysis of the sampling variance was significant (*p* < 0.05). Nevertheless, the trim and fill analysis did not reveal any missing effect sizes, suggesting that there was no indication of publication bias.

For HR/HRV, the Rosenthal fail‐safe test demonstrated that there was no indication of publication bias. Moreover, the funnel plot appeared to be symmetrical and the regression analysis of the sampling variance was not significant (*p* = 0.801). Likewise, the trim and fill analysis revealed no missing effects sizes, suggesting that there was no indication of publication bias.

### 3.8 Moderator analyses

Tables 3 and 4 report the results of the moderator analyses on cardiovascular (i.e., BP and HR/HRV) parameters of stress. Only significant results are described here.

**TABLE 3:** Results for the moderator analyses on blood pressure

Moderator	*N* samples	*N* ES	*B*_0_ (95% CI)	*t*_0_	*B*_1_ (95% CI)	*t*_1_	*F* (df_1_, df_2_)	*p*
*Intervention characteristics*								
Component mindfulness							*F* (1, 82) = 46.824	<0.001
Yes (RC)	6	52	0.859 (0.489 to 1.230)	4.619***				
No	10	32	−0.335 (−0.663 to 0.008)	−2.038*	−1.195 (−1.542 to 0.848)	−6.843***		
Component relaxation							*F* (1, 82) = 44.734	<0.001
Yes (RC)	11	66	0.531 (0.151 to 0.911)	2.778**				
No	5	18	−0.702 (−1.134 to 0.269)	−3.227**	−1.233 (−1.599 to 0.866)	−6.693***		
Component yoga							*F* (1, 82) = 8.617	0.004
Yes (RC)	3	14	−0.271 (−0.602 to 0.061)	−1.623		2.936**		
No	13	70	0.254 (0.125 to 0.383)	3.925***	0.525 (0.169 to 0.881)			
Component cognitive‐behavioural							*F* (1, 82) = 44.734	<0.001
Yes (RC)	8	36	−0.463 (−0.866 to 0.060)	−2.285*				
No	8	48	0.757 (0.344 to 1.170)	3.647***	1.220 (0.857 to 1.583)	6.688***		
Intensity (continuous)	16	84	0.209 (−0.211 to 0.629)	0.989	0.001 (0.001 to 0.001)	6.322***	*F* (1, 82) = 39.972	<0.001
*Sample characteristics*								
Target group							*F* (1, 82) = 0.862	0.356
Selected (RC)	5	36	0.252 (0.041 to 0.463)	2.372*				
Non‐selected	11	48	0.124 (−0.050 to 0.298)	1.414	−0.128 (−0.402 to 0.146)	−0.928		
% Boys (continuous)	15	80	0.180 (0.035 to 0.324)	2.467*	−0.002 (−0.010 to 0.006)	−0.409	*F* (1, 78) = 0.167	0.684
% Minorities (continuous)	12	74	0.190 (0.052 to 0.327)	2.754**	0.005 (−0.000 to 0.011)	1.838	*F* (1, 72) = 3.379	0.070
Mean age (continuous)	16	84	0.180 (0.057 to 0.304)	2.906**	0.063 (−0.002 to 0.128)	1.916	*F* (1, 82) = 3.672	0.059
*Study characteristics*								
Type of BP measurement							*F* (1, 82) = 1.186	0.279
SBP (RC)	16	42	0.241 (0.056 to 0.425)	2.591*				
DBP	16	42	0.106 (−0.079 to 0.290)	1.138	−0.135 (−0.381 to 0.112)	−1.089		
Stress outcome measure							*F* (1, 81) = 2.042	0.136
Basal functioning (RC)	12	28	0.132 (−0.082 to 0.346)	1.227				
Ambulatory monitoring	5	42	0.286 (0.116 to 0.455)	3.354**	0.154 (−0.119 to 0.427)	1.120		
Stress reactivity	3	14	−0.076 (−0.407 to 0.256)	−0.453	−0.208 (−0.602 to 0.187)	−1.046		
Publication year (continuous)	16	84	0.169 (0.026 to 0.313)	2.346*	−0.001 (−0.017 to 0.015)	−0.121	*F* (1, 82) = 0.015	0.904
Study design							*F* (1, 82) = 5.488	0.022
(Cluster) RCT (RC)	14	80	0.212 (0.082–0.342)	3.235**				
Quasi‐experimental	2	4	−0.521 (−1.130 to 0.088)	−1.703	−0.733 (−1.356 to 0.111)	−2.343*		
Comparison condition							*F* (1, 82) = 0.986	0.324
Passive control (RC)	6	16	0.048 (−0.240 to 0.336)	0.333				
Active control	10	68	0.211 (0.058 to 0.364)	2.743**	0.163 (−0.163 to 0.489)	0.993		
Timing of outcome measurement							*F* (1, 82) = 0.605	0.439
Post‐intervention	10	58	0.198 (0.032 to 0.364)	2.371*				
Follow‐up	6	22	0.085 (−0.169 to 0.340)	0.667	−0.112 (−0.400 to 0.175)	−0.778		
Study quality (continuous)	16	84	0.176 (0.036 to 0.316)	2.505*	0.022 (−0.033 to 0.077)	0.801	*F* (1, 82) = 0.642	0.425

Abbreviations: *B*_0_, mean effect size Cohen's *d*; BP, blood pressure; CI, confidence interval; *B*_1_, estimated regression coefficient; DBP, diastolic blood pressure; *F*‐value, omnibus test of regression coefficients; *N* ES, number of effect sizes; *N* samples, number of independent samples; *p*, *p*‐value of omnibus test; RC, reference category; RCT, randomized controlled trial; SBP, systolic blood pressure; *t*‐values, difference in mean *d* with zero

*p < 0.05; **p < 0.01; ***p < 0.001.

**TABLE 4:** Results for the moderator analyses on heart rate

Moderator	*N* samples	*N* ES	*B*_0_ (95% CI)	*t*_0_	*B*_1_ (95% CI)	*t*_1_	*F* (df_1_, df_2_)	*p*
*Intervention characteristics*								
Component mindfulness							*F* (1, 59) = 4.219	0.044
Yes (RC)	7	28	0.391 (0.059 to 0.723)	2.359*				
No	13	33	−0.030 (−0.316 to 0.255)	−0.212	−0.421 (−0.832 to 0.011)	−2.054*		
Component relaxation							*F* (1, 59) = 1.130	0.292
Yes (RC)	12	36	0.230 (−0.051 to 0.510)	1.639				
No	8	25	0.012 (−0.318 to 0.342)	0.072	−0.218 (−0.628 to 0.192)	−1.063		
Component yoga							*F* (1, 59) = 0.083	0.775
Yes (RC)	6	16	0.187 (−0.235 to 0.610)	0.887				
No	14	45	0.117 (−0.133 to 0.367)	0.937	−0.071 (−0.561 to 0.420)	−0.561		
Component cognitive‐behavioural							*F* (1, 59) = 5.414	0.023
Yes (RC)	11	30	−0.066 (−0.341 to 0.209)	−0.481				
No	9	31	0.382 (0.084 to 0.680)	2.569*	0.448 (0.063 to 0.834)	2.327*		
Intensity (continuous)	20	61	0.132 (−0.071 to 0.335)	1.298	0.000 (0.000 to 0.001)	2.999**	*F* (1, 59) = 8.996	0.004
*Sample characteristics*								
Target group							*F* (1, 59) = 1.084	0.302
Selected (RC)	8	29	0.010 (−0.299 to 0.320)	0.068				
Non‐selected	12	32	0.226 (−0.049 to 0.501)	1.645	0.216 (−0.199 to 0.630)	1.041		
% Boys (continuous)	18	58	0.133 (−0.094 to 0.360)	1.178	−0.005 (−0.018 to 0.007)	−0.814	*F* (1, 56) = 0.663	0.419
% Minorities (continuous)	14	48	0.139 (−0.112 to 0.389)	1.114	−0.007 (−0.016 to 0.001)	2.895	*F* (1, 46) = 2.895	0.096
Mean age (continuous)	20	61	0.136 (−0.080 to 0.352)	1.259	0.005 (−0.111 to 0.121)	0.085	*F* (1, 59) = 0.007	0.932
*Study characteristics*								
Type of HR measurement							*F* (1, 59) = 0.078	0.781
Heart or pulse rate (RC)	19	46	0.121 (−0.112 to 0.353)	1.039				
HRV	4	15	0.181 (−0.218 to 0.580)	0.908	0.060 (−0.371 to 0.492)	0.279		
Stress outcome measure							*F* (1, 59) = 0.739	0.482
Basal functioning (RC)	16	27	0.189 (−0.081 to 0.458)	1.402				
Ambulatory monitoring	4	15	−0.098 (−0.525 to 0.329)	−0.459	−0.287 (−0.786 to 0.212)	−1.149		
Stress reactivity	6	19	0.204 (−0.150 to 0.557)	1.153	0.015 (−0.394 to 0.423)	0.073		
Publication year (continuous)	20	61	0.123 (−0.088 to 0.334)	1.164	−0.013 (−0.039 to 0.013)	−0.969	*F* (1, 59) = 0.938	0.337
Study design							*F* (1, 59) = 0.028	0.867
(Cluster) RCT (RC)	17	54	0.128 (−0.103 to 0.358)	1.109				
Quasi‐experimental	3	7	0.181 (−0.406 to 0.768)	0.617	0.053 (−0.578 to 0.684)	0.168		
Comparison condition							*F* (1, 59) = 0.011	0.916
Passive control (RC)	9	30	0.124 (−0.186 to 0.434)	0.798				
Active control	11	31	0.147 (−0.154 to 0.448)	0.975	0.023 (−0.409 to 0.455)	0.106		
Timing of outcome measurement							*F* (1, 59) = 1.372	0.246
Post‐intervention (RC)	15	54	0.094 (−0.131 to 0.318)	0.834				
Follow‐up	5	7	0.387 (−0.093 to 0.867)	1.614	0.294 (−0.208 to 0.795)	1.172		
Study quality (RC)	20	61	0.128 (−0.081 to 0.337)	1.228	−0.035 (−0.105 to 0.035)	−1.001	*F* (1, 59) = 1.002	0.321

Abbreviations: *B*_0_, mean effect size Cohen's *d*; *B*_1_, estimated regression coefficient; CI, confidence interval; *F*‐value, omnibus test of regression coefficients; HRV, heart rate variability; *N* ES, number of effect sizes; *N* samples, number of independent samples; *p*, *p*‐value of omnibus test; RC, reference category; RCT, randomized controlled trial; *t*‐values, difference in mean *d* with zero.

*p < 0.05; **p < 0.01; ***p < 0.001.

*Intervention characteristics*. For BP, components and intensity moderated the effects. Larger effects were found for intervention programs with a mindfulness and/or meditation component compared to programs without this component and for interventions with a relaxation component versus interventions without a relaxation component. Interventions with a yoga component were not effective, as opposed to interventions without a yoga component. Smaller effects were observed for intervention programs with a cognitive‐behavioural component versus interventions without this component. Additionally, larger effects were found for interventions with a higher intensity. For HR/HRV, significant positive effects were found for intervention programs with a mindfulness and/or meditation component, but not for intervention programs without this component. Furthermore, interventions with a cognitive‐behavioural component were not effective compared to interventions without this component. In addition, larger effects were observed for interventions with a higher intensity.

*Sample characteristics*. There were no significant moderators for HR/HRV and BP.

*Study characteristics*. For BP, significant effects were found for (cluster) RCTs, but not for quasi‐experimental designs.

## 4 DISCUSSION

In the current study, we examined the effectiveness of school‐based intervention programs in improving HPA‐axis (i.e., cortisol) and cardiovascular (i.e., SBP/DBP and HR/HRV) parameters of stress in adolescents. For BP and HR/HRV, multilevel meta‐analyses were conducted to investigate the effectiveness of school‐based intervention programs on cardiovascular parameters of stress. This multilevel approach is favourable, as it takes into account the dependency of multiple effect sizes from the same study, allowing the inclusion of all relevant effect sizes and thus generate more power (Assink & Wibbelink, 2016). Results demonstrated that school‐based intervention programs had a small overall effect on reducing BP in adolescents, while the overall effect for HR/HRV was not significant. For cortisol, we did not analyse the data using a multilevel meta‐analysis due to the large variation in methodology in the small number of included primary studies. An overview of the findings of the included studies showed that cortisol results were inconsistent, likely caused by the large methodological variation. Additionally, we investigated intervention (i.e., components, intensity), sample (i.e., target group, gender, minority background, and age), and study (i.e., type of HR and BP measurement, type of stress outcome, publication year, study design, type of comparison condition, timing of outcome measurement, and study quality) characteristics as moderators of effectiveness. For BP and HR/HRV, several moderators influenced the effectiveness of school‐based intervention programs. Larger effects were observed for intervention programs with a mindfulness and/or meditation component, for interventions without a cognitive‐behavioural component and for interventions with a higher intensity. For BP, significant program effects were only demonstrated in (cluster) RCTs and not in studies with a quasi‐experimental design. Also for BP only, larger effects were found for intervention programs with a relaxation component while programs with a yoga component were not effective compared to programs without a yoga component.

The overall finding that intervention programs are effective in improving BP is consistent with previous literature that showed improved cardiovascular parameters of stress in community and clinical samples comprising mainly adults (Heckenberg et al., 2018; Pascoe & Bauer, 2015; Pascoe, Thompson, Jenkins, et al., 2017; Pascoe, Thompson, & Ski, 2017). As such, the present study demonstrates that indicators of physiological stress (i.e., SBP and DBP) are also malleable by intervention programs in adolescents. In the current study, both cardiovascular parameters of stress demonstrated improvements after school‐based intervention programs, although only BP showed a significant improvement. This indicates that BP might be a more susceptible target for school‐based intervention programs than HR/HRV. A previous study in healthy adults also observed significant reductions in BP, but not HR, after a slow pace breathing exercise (Pramanik et al., 2009). Likewise, another study demonstrated larger effects for SBP as opposed to HR after slow, deep breathing in patients with hypertension (Bhavanani et al., 2011), suggesting that BP might be more sensitive to change. Thus, BP seems to be an important parameter to include when studying the effectiveness of interventions to reduce stress in adolescents. That said, the absence of a significant overall effect for HR/HRV is in contrast with most previous findings based on mainly adult samples (Heckenberg et al., 2018; Pascoe & Bauer, 2015; Pascoe, Thompson, Jenkins, et al., 2017; Pascoe, Thompson, & Ski, 2017). This may suggest developmental differences in responsiveness to intervention programs targeting stress reduction, at least with respect to HR/HRV, that require further examination in future research. Alternatively, a methodological explanation for the non‐significant overall effect for HR/HRV could be the specific intervention programs examined in relation to HR/HRV compared to BP. Characteristics of these programs (i.e., less intensive, less often consisted a mindfulness and/or meditation component), had smaller effects in the current study, and may therefore explain the diverging findings for HR/HRV. In addition, the previous meta‐analytic studies that demonstrated improved HR/HRV focused predominantly on mindfulness and meditation programs (Heckenberg et al., 2018; Pascoe, Thompson, Jenkins, et al., 2017; Pascoe, Thompson, & Ski, 2017). Finally, the lack of a significant overall effect for HR/HRV might be the result of the smaller number of included effect sizes for this indicator, resulting in less statistical power to detect significant effects.

In the relatively small number of studies with cortisol measurement included in this review, HPA‐axis functioning was measured in different ways, with variations in sample collection, number of samples, timing of sample collection, and analytic approach. Because this hampers comparability, no meta‐analysis was performed for this outcome. Across the nine studies and ways of cortisol measurements, findings were inconsistent. Furthermore, the studies that showed significant intervention effects did not notably differ from the studies that demonstrated no effects (e.g., in methodology, type of intervention program). In a recent meta‐analysis investigating the effects of mindfulness‐based interventions on cortisol in healthy adults, it was also observed that results might have been influenced by variation in the assessment of cortisol, including sample collection strategy, total days of measurements, and indicators of assessment (Sanada et al., 2016). Salivary cortisol levels are often used as an indicator of stress, especially in children and adolescents, because the collection of salivary samples is relatively easy and noninvasive, as well as inexpensive (Hanrahan et al., 2006). However, it must be taken into account that many factors can influence the measurement of salivary cortisol. Previous research showed that the time and location of sampling, type of assay used, and units of measurements might impact the accuracy and reliability of measurements. Future studies should take into account this complexity of cortisol measurements, and make sure that uniform procedures and methods are used to obtain cortisol levels, and that the data‐collection is well documented. Before incorporating measurements of salivary cortisol, researchers must develop a rigorous protocol for sample collection, including standardizing the time for sample collection and using multiple days of measurements (Hanrahan et al., 2006; Sanada et al., 2016). In this way, results will be more comparable across studies and it will be possible to draw more accurate conclusions.

Moderator analyses demonstrated that for both BP and HR/HRV, larger effects were observed for intervention programs with a mindfulness and/or meditation component and smaller effects were found for intervention programs with a cognitive‐behavioural component. For BP only, larger effects were found for intervention programs with a relaxation component. These findings contradict results of a previous meta‐analysis examining the effectiveness of school programs for children and adolescents (Kraag et al., 2006), demonstrating larger effects for components to enhance problem solving and emotional coping skills compared to relaxation techniques. However, results of Kraag et al. (2006) were based on a small number of studies, examined only universal interventions and mainly reflected psychological stress. Hypothetically, cognitive‐behavioural techniques may have more impact on psychological stress, and techniques as mindfulness, meditation, and relaxation might be more effective in improving physiological parameters of stress. Although the neurobiological effects of mindfulness, meditation, and relaxation techniques are not yet clearly understood, recent reviews provide evidence that they are associated with biological changes in BP and HR (Pascoe & Crewther, 2016; Pascoe, Thompson, Jenkins, et al., 2017), specifically in programs that focus on breathing (Pascoe & Crewther, 2016). A homeostatic state is characterized by relaxed breathing, and since breathing is one of the actions of the ANS that can be individually controlled, it is plausible that such techniques have more impact on improving physiological parameters of stress than cognitive‐behavioural techniques (Pascoe & Crewther, 2016). Another explanation could be that some intervention programs, especially programs with a cognitive‐behavioural component, first lead to changes in psychological stress, and only after some additional time lead to changes in physiological stress. This suggests that short‐term effects (i.e., post‐intervention) may be less likely and smaller than long‐term effects (i.e., follow‐up). However, moderator analyses did not confirm this line of reasoning, as there were no differences between post‐intervention and follow‐up effects. Unfortunately, only few studies included follow‐up measurements. To draw accurate conclusions about long‐term effects on physiological parameters of stress, it is necessary that future intervention studies include both post‐intervention and follow‐up measurements. Previous research demonstrated that perceived stress reflects activity of the HPA‐axis as well as the ANS, but only to a certain extent (Oldehinkel et al., 2011), which may explain diverging findings in terms of psychological and physiological stress. Since few intervention studies measure both psychological and physiological stress, future studies should investigate the mutual relationship between both indicators of stress, to discover their association over time. Finally, in the current study intervention programs with a cognitive‐behavioural component often reflected broad and generic programs (e.g., stress management as a small part of the program, or adolescents were taught general coping skills). As such, stress reduction was often a secondary or indirect program aim, which might explain the observed smaller effects for intervention programs with a cognitive‐behavioural component.

For BP only, significant effects were only observed for programs without a yoga component, and not for programs with a yoga component. This contradicts earlier research, which demonstrated that yoga practices improved regulation of the SNS (Pascoe & Bauer, 2015; Pascoe, Thompson, & Ski, 2017). However, as these results were observed in predominantly adult samples, it is possible that yoga interventions are not or less effective for adolescents in reducing physiological parameters of stress. Indeed, a previous study demonstrated that adolescents with irritable bowel syndrome benefitted less from a yoga intervention than young adults with this disorder (Evans et al., 2014), possibly related to the challenge of motivating adolescents. Future research should investigate these potential developmental differences in response to yoga interventions. Alternatively, it could be that variations in focus of yoga‐interventions, such as physical postures, controlled breathing or meditation techniques, accounted for the results. Since programs that focus on breathing have been proven beneficial (Pascoe & Crewther, 2016), it is plausible that yoga programs that only involve physical postures yield smaller effects on physiological parameters of stress than programs with a predominant focus on controlled breathing and meditation. Indeed, the included studies with a yoga component (i.e., examining BP) consisted of both physical postures and controlled breathing or meditation, with physical postures as the main focus. Nevertheless, this finding should be interpreted with caution, because it was based on a small sample (i.e., 3 studies, 14 effect sizes).

For BP and HR/HRV, larger effects were observed for intervention programs with a higher intensity. Visual inspection of the data showed that interventions with an intensity of 1000 min or more (at least 17 h) were more beneficial (i.e., generated higher effect sizes). This is in contrast with Stice et al. (2009), demonstrating larger effects on depressive symptoms for prevention (i.e., coping skills) programs for children and adolescents with shorter durations. It is possible that higher intensity programs are necessary to improve physiological parameters of stress, whereas psychological effects of stress are more easily (i.e., faster) obtained, and thus require less intensive programs. In fact, in the present study, smaller effects were observed for intervention programs with a cognitive‐behavioural component, and these programs were less intensive than programs without a cognitive‐behavioural component. Accordingly, the importance of program intensity might correlate with the type of intervention program. Mindfulness, meditation, and relaxation techniques may need longer periods of time to master than cognitive‐behavioural techniques. Hence, mindfulness, meditation, and relaxation programs that are more intensive might be more effective in improving physiological parameters of stress. Indeed, a recent review demonstrated that programs involving more hours of meditation seemed to be more beneficial than programs with fewer meditation hours (Pascoe & Crewther, 2016).

Finding only significant effects on BP for (cluster) RCTs and not for quasi‐experimental designs contradicts a previous meta‐analysis that investigated the effectiveness of interventions for children and adolescents with emotional and behavioural disorders (Suter & Bruns, 2009). In this meta‐analysis, larger effects were demonstrated for studies with a quasi‐experimental design versus RCTs, although methodological factors other than treatment allocation may have influenced the results (Suter & Bruns, 2009). Besides these considerations, the current finding that (cluster) RCTs are more effective should be interpreted with caution, because only two studies examining BP were quasi‐experimental (i.e., four effect sizes).

## 5 LIMITATIONS AND RECOMMENDATIONS FOR FUTURE RESEARCH

Some limitations need to be considered in this review. First, limited information was available for some of the study, sample, and intervention characteristics, including study population (i.e., rural or urban), percentage of socio‐economic status, and program integrity. This lack of information prevented us from conducting moderator analyses. In order to determine what characteristics are beneficial and which programs work for specific subsamples, we recommend future intervention studies to report sufficient information about study, sample, and intervention characteristics. For example, program integrity is very important to take into account, because non‐significant or negative results might be due to incorrect program implementation instead of an ineffective program (Lane et al., 2004).

Second, methodological differences were observed for assessments of physiological parameters of stress, especially for sampling of cortisol. Differences were observed in sample collection, timing of sample collection, number of samples, and approach of analysing the data. Future studies should be aware of methodological differences and provide detailed protocols about their data collection to account for this.

Third, studies were included that measured basal functioning, stress reactivity, and ambulatory monitoring. Although there was no significant moderator effect demonstrated for these different measures, it is possible that this influenced the effects. Also, only a small number of studies, and for cortisol not a single study, included stress reactivity. Yet, it is important that future studies also investigate reactivity, to further assess the potential of stress reduction programs to improve stress reactivity. However, as cardiovascular parameters of stress (i.e., HR and BP) are affected immediately and throughout stress exposure, while cortisol takes longer to reach peak levels (i.e., post‐stress) (Allen et al., 2014; Linden et al., 1997), it is important to take these differences into account by measuring at multiple timepoints. Additionally, only some of our included studies measured HRV (i.e., 25% of the ES). Since HRV is a reliable indicator of ANS activity related to stress (Kim et al., 2018), we encourage future studies to include HRV measurements as physiological stress outcomes.

Fourth, only few studies included follow‐up measurements. By including long‐term assessments, potential sleeper effects (i.e., improved longer term outcomes) could be observed. For instance, previous research of an 8‐weeks mindfulness intervention in cancer patients demonstrated linear salivary cortisol decreases over the course of the study (i.e., post‐intervention, 6‐months follow‐up, and 12‐months follow‐up). Yet, linear decreases were not observed for BP and HR (Carlson et al., 2007), suggesting that the timing of physiological effects of intervention programs differs across parameters. Further research in this area is needed to advance our understanding of the timing and sequencing of intervention effects across physiological parameters of stress.

Finally, although we found no significant differences between active and passive control conditions, one should be aware that the type of control condition can influence effect estimates of psychosocial interventions (Karlsson & Bergmark, 2015). As such, type of control condition is crucial to take into consideration when interpreting intervention effects.

Overall, to increase the knowledge on physiological effects of (school‐based) intervention programs, it is crucial that researchers conduct physiological measurements in a more uniform way to improve comparability of future studies. In this way, researchers will be able to systematically determine the role of physiological stress parameters and their change as a result of intervention programs, demonstrating which programs are most beneficial. In addition, preferably, researchers should include participants from various age groups (e.g., children, adolescents, adults) and educational levels, include both physiological and psychological stress outcomes, and include measurements immediately after the intervention as well as follow‐up measurements after completion of the intervention.

## 6 CONCLUSIONS

The present review demonstrated that school‐based intervention programs promoting psychosocial functioning have the potential to improve ANS indicators of stress. Since chronic stress is a mental health issue among adolescents (Walburg, 2014) and a dysregulated stress system is associated with mental health problems (Charmandari et al., 2005), it is important to address heightened stress levels in adolescents and give them tools to adequately cope with stress. Previous research focused mainly on psychological stress, demonstrating that school‐based intervention programs reduce adolescent psychological stress (Kraag et al., 2006; van Loon et al., 2020). The current study is a valuable addition to the literature as it demonstrates that school‐based intervention programs promoting psychosocial functioning have the potential to also improve physiological parameters of stress. Future researchers should, therefore, add physiological outcomes in studies on the effectiveness of stress reducing programs. Moreover, governments and schools should be aware of the availability of such school‐based intervention programs, and implement them. Particularly, to improve functioning of the ANS, one of the major stress systems, intervention programs should include a mindfulness, meditation or relaxation component, since these components were most effective. In addition, programs with a higher intensity should be encouraged. Providing adolescents with techniques to reduce physiological stress through school‐based intervention programs may prevent emerging mental health problems.

## CONFLICTS OF INTEREST

The authors declare that they have no conflicts of interest.

## AUTHOR CONTRIBUTIONS

Amanda W. G. van Loon conceived and participated in the design of the study, conducted the search and extracted data, performed data analysis and drafted the manuscript. Hanneke E. Creemers conceived and participated in the design of the study, coordinated the study and drafted the manuscript. Ana Okorn participated in data collection and interpretation of the study, and critically revised the manuscript. Simone Vogelaar participated in interpretation of the data and critically revised the manuscript. Anne C. Miers participated in the interpretation of the data and critically revised the manuscript. Nadira Saab participated in the interpretation of the data and critically revised the manuscript. P. Michiel Westenberg participated in the interpretation of the data and critically revised the manuscript. Jessica J. Asscher conceived and participated in the design of the study, coordinated the study and drafted the manuscript. All authors read and approved the final manuscript.

## Data Availability

The datasets generated and analysed during the current study are available from the corresponding author on reasonable request.
